# Biomechanical analysis of different osteosynthesis configurations in the pin and plate fixation method for distal humerus fractures

**DOI:** 10.1186/s12891-023-06709-y

**Published:** 2023-07-17

**Authors:** Alireza Hakiminejad, Amir Nourani, Narges Ghias, Alireza Mahmoudi, Kaveh Same, Reza Shahriar Kamrani, Mohammad Hossein Nabian

**Affiliations:** 1 Department of Mechanical Engineering, Sharif University of Technology, Tehran, Iran; 2 School of Medicine, Tehran University of Medical Sciences, Tehran, Iran; 3 Department of Orthopedic and Trauma Surgery, Tehran University of Medical Sciences, Tehran, Iran; 4 Center of Orthopedic Trans-Disciplinary Applied Research (COTAR), Tehran University of Medical Sciences, Tehran, Iran

**Keywords:** Persian fixation method, Distal humerus fracture, Stability, Biomechanical testing, Osteosynthesis

## Abstract

Distal humerus fractures commonly occur in adults with low bone mineral density causing major technical challenges for orthopedic surgeons. Persian fixation method was introduced as a novel technique to stabilize small fragments in comminuted distal humerus fractures using a set of K-wires and a reconstruction plate. The present study aims to measure this technique's stiffness and stability of this technique and analyze the effect of influential parameters with numerical simulation and biomechanical testing on a cadaveric specimen. Validation of the finite element (FE) model was conducted based on results of experiments. The results indicated that Delta configuration mainly led to a higher stiffness in the case of axial loading and anterior bending compared to L configuration. Analyzing the influential factors of this technique suggests that changes in diameter and number of K-wires have a similarly significant effect on the construct stiffness while the height of plate had a slight influence. Also, the diameter of wires was the most effective parameter for implant failure, particularly in the 3-pin construct, which caused a reduction in failure risk by about 60%. The results revealed that the Persian fixation method would achieve suitable stability compared to the dual-plating technique.

**Supplementary information:**

The online version contains supplementary material available at 10.1186/s12891-023-06709-y.

## Introduction

Fractures of distal humerus include more than 2% of all fractures occurring in adults with osteoporosis and are escalating due to low bone mineral density caused by raising the average age of communities [1–3]. Bone fragility causes various complex fractures in the distal humerus posing a technical challenge in orthopedic surgery [4]. Different approaches have been proposed for the treatment of distal humeral fractures. The main purpose of fracture treatment is to restore complex anatomical form and recover the functional range of elbow movement, which can be achieved using a rigid fracture fixation [5–7]. Non-operative or conservative treatment involving closed reduction along with splinting techniques generally led to unacceptable outcomes on account of an inadequate reduction and imprecise restoration of the articular surface of humeral anatomy [8]. Hence, open reduction and internal fixation (ORIF) seems feasible, but it is also prone to different complications [8]. Despite the considerable evolution of surgical treatments for distal humerus fractures, the literature indicates an undesirable post-surgery outcome of about 20–25% that can be minimized by choosing the right approach of treatment [9].

Currently, the plate and screw fixation is a common choice for orthopedic surgeons to stabilize distal humerus fractures. The majority of the studies were conducted on the importance of plates number using two plates to ensure adequate rigidity of fixation and it is broadly confirmed by surgeons [10]. Double-plate systems can be arranged in parallel or perpendicular configurations. To date, many studies have been done on biomechanical aspects of these methods with rare clinical reports on them. An experimental in-vitro study aimed at comparing two configurations did not observe statistical differences between them [11]. Also, previous clinical studies [12, 13] observed no significant difference between these configurations regarding clinical outcomes; e.g., bony union of all patients treated with these methods occurred in an average of 6 months after surgery and they resulted in almost a similar range of motion (ROM) in both flexion and extension according to the last follow-up [12, 13]. Nevertheless, the parallel configuration was shown in some studies to provide better biomechanical properties [14–16].

Other techniques such as Kirschner-wires have also been utilized to fix distal humerus fractures; i.e. the fragments were kept in anatomical position by the inserted K-wires [17]. Furthermore, this method of fixation was an acceptable choice for the treatment of pediatric supracondylar fractures, though it did not provide sufficient stability for the treatment of distal humeral fractures in adults [18].

Each of mentioned methods had specific uses for the treatment of fractures, while some features were undesirable. The thickness of plates caused difficulty contouring for adjusting to bone surfaces, resulting in a lower stability in distal portions especially in low supracondylar fractures with small pieces. In such cases, most ORIF methods are not practical, and, therefore, using an elbow prosthesis would remain as the only choice for treatment [17]. In 2011, Kamrani et al. [19] proposed Persian fixation method as a novel technique to stabilize complex and low supracondylar distal humerus fractures. This method utilizes a set of smooth Kircshner wires with reconstruction plates that can be performed in different configurations. In this procedure, K-wires used to keep the distal fragments in the anatomical position are bent from the medial and lateral sides and placed under the reconstruction plate on humeral shaft at a distance from the wire insertion area. This technique was used to treat low distal humerus fractures involving the articular surface for nineteen patients with poor bone qualities and an average age of 46 (range, 17–73). The patients were followed up at 6-week periods for an average of 12 months; the elbow ROM and heterotopic ossification were recorded, and stability of elbow was tracked by reviewing the radiographs for displacement and union. The average ROM was ${115}^{^\circ }$ and ${16}^{^\circ }$ in flexion and extension, respectively, with the average total arc of ${99}^{^\circ }$ [19]. The Mayo elbow performance index ranged from 60–100 with an average of 88, which is considered a good score according to rating systems for evaluation of the elbow established by Longo et al. [20]. Hence, Persian fixation method is a suitable method for complex fractures based on clinical outcomes reported. This technique can be used in comminuted and low supracondylar fractures, which are difficult or even impossible to be stabilized with other fixation methods [19].

Despite of some promising clinical observations, the biomechanical properties of Persian fixation method have not been studied so far; i.e. the strength and stiffness of this approach have not been measured, and the parameters involved have not been investigated in a controlled manner. The present study aims to measure the stiffness and strength of the Persian fixation method using the finite element method (FEM) and biomechanical testing.

## Material and methods

### Methodology

**Fig. 1:**
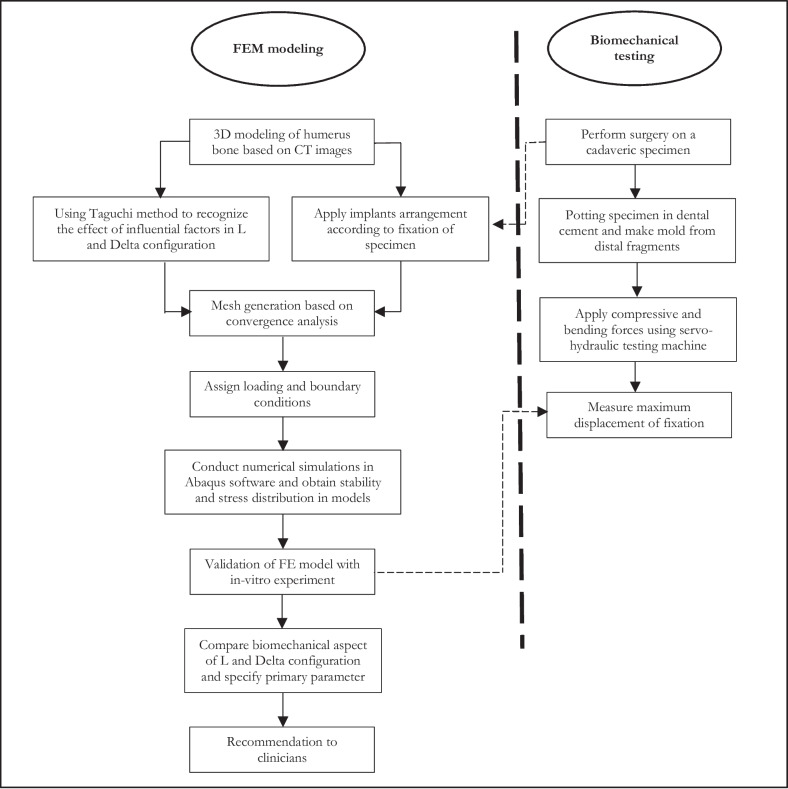
Research methodology for analyzing Persian fixation method.

The Flowchart diagram on this study's methodology is shown in Fig. 1. In the numerical simulation, the effective parameters in the arrangement were analyzed under axial and anterior bending loading. Experiments for both L and Delta arrangements were designed to examine the effects of involving parameters (e.g., diameter and the number of wires along with the height of the reconstruction plate) on the biomechanical response of the fixation. In order to validate the results of numerical simulations, biomechanical testing on a cadaveric specimen was performed. We hypothesized that by detecting the most effective parameters in this technique, a fixation with rigidity similar to that of the dual plating method can be achieved.

### D modelling

A 3D model of the right humerus of a 32-year-old male was generated based on high-quality CT images (NeuViz 16, 16-slice Computed Tomography Scanner). The DICOM format of CT images with increments of 1 mm was imported into a self-development image processing software. The segmentation mask of bone tissue was made by selecting the threshold between 226–3171 (Hounsfield unit). At last, an STL file of the 3D model was created.

### Design of experiments (DoE)

**Fig. 2:**
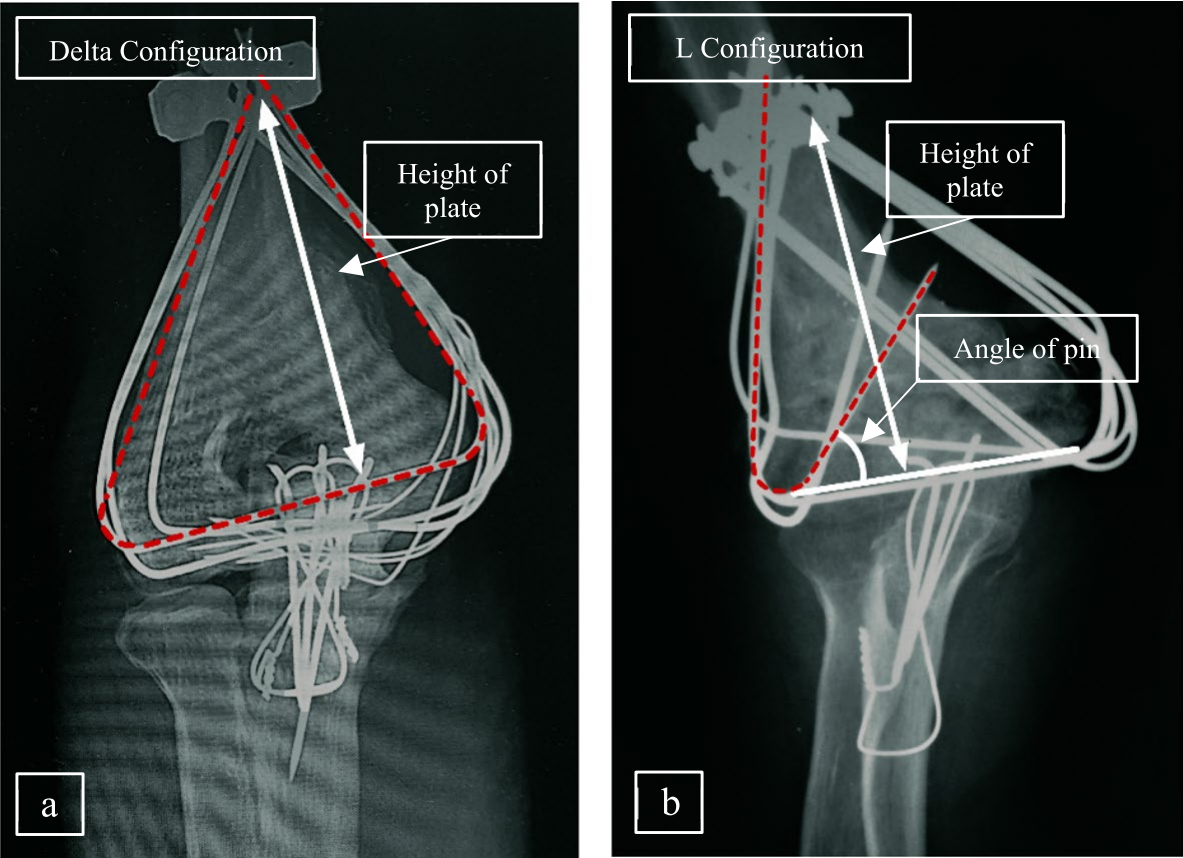
Anteroposterior radiograph images of (**a**) Delta and (**b**) L configurations.

Two different configurations of implants in the Persian fixation method (i.e. L and Delta arrangements) are shown in Fig. 2. For each fixation arrangement, design of experiments was performed. The parameters considered for DoE were specified according to clinical experiences by senior surgeons.

To achieve an optimal number of experiments for Delta and L configurations, the Taguchi and full-factorial methods were employed, respectively, as listed in Table 1 and Table 2. In DoE for Delta arrangement, three influential parameters with three levels were considered: a) number of pins; 1 to 3 wires can be located in the proximal–distal direction, b) diameter of pins; the diameter of each wire may be selected 1, 1.5, or 2 mm, c) fixation height of the reconstruction plate; due to the 25 cm length of each pin, fixation height was assumed in the range of 55–75 mm (Fig. 2) with a 10-mm step between different levels.

**Table 1:** Design of experiments for Delta configuration using Taguchi method

Run order	Number of wires	Diameter of wires (mm)	Height of plate (mm)
1	1	1	55
2	1	1.5	65
3	1	2	75
4	2	1	65
5	2	1.5	75
6	2	2	55
7	3	1	75
8	3	1.5	55
9	3	2	65

**Table 2:** Design of experiments for L configuration using full-factorial method

Run order	Angle of wires (°)	Height of plate (mm)
10	0	60
11	0	110
12	30	60
13	30	110
14	60	60
15	60	110

In case of L configuration, the results obtained from Delta configuration were used for DoE to reduce the number of simulations; i.e. since the maximum number of wires with the largest diameter yielded the best results (see Sect. 3.1), three K-wires, each having a diameter of 2 mm, were applied in lateral and medial sides. In this case, the plate fixation height had a broader range with respect to that for Delta configuration because of one side egression from fragments, i.e. it was defined in two levels of 60 and 110 mm. The angle that the pin made with the epicondylar axis (see Fig. 2) was considered in 3 levels (0, 30 or 60°). The full factorial design for the L method is given in Table 2.

### Finite element modeling

In the humerus bone, cancellous bone was surrounded by cortical bone with a thickness of 2.5 mm, referring to CT images. The AO 13C1.1 fracture (AO Foundation, Davos, Switzerland) with Y-shape intra-articular fracture was reproduced in the CAD models to simulate a simple articular fracture above the epicondylar axis [21]. No gaps were considered between fragments to mimic the best condition for achieving acceptable anatomical reduction during a surgical operation, as recommended by the surgeons who participated in the current study. Furthermore, presence of contact between the distal and proximal fragments was necessary for contouring wires during surgery to form suitable rigidity in the construct.

The properties of 316L AISI stainless austenitic steel were assigned to Kirschner wires; i.e. Young’s modulus of 200 GPa and Poisson’s ratio of 0.3 [22]. Young’s moduli assigned for cortical and trabecular bone were 20 and 0.5 GPa, respectively, with Poisson’s ratio of 0.3 for both segments [23].

**Fig. 3:**
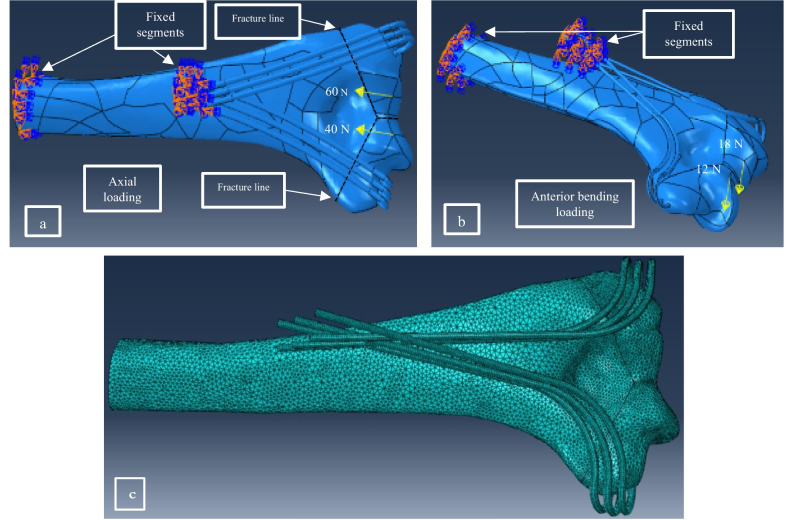
The loading and boundary conditions of FE models: (**a**) axial compressive loading, (**b**) anterior bending loading, (**c**) 3D meshing in FE model.

The loading and boundary conditions for numerical simulations are illustrated in Fig. 3. The compressive load of 100 N in total was applied at the distal fragments with a distribution of 60% at the lateral and 40% at the medial (Figure 3a) [11, 14, 22, 24]. The anterior bending was applied with similar distribution on the ulna and radial column by a 30 N bending force perpendicular to the axis of the humerus diaphysis (Fig. 3b) [11, 14, 22, 24].

Fixed boundary conditions in the FEM were defined for the proximal end of the humerus bone. K-wires were fixed on the humeral diaphysis to simulate the constraint imposed by the plate. Surface interactions were defined by applying a general contact with coefficients of friction of 0.3 and 0.6 in the bone-steel and bone-bone interfaces, respectively [25, 26]. Also, a tie constraint was applied between the cortical and cancellous bones in different parts of the models. To evaluate the precision of the models, a mesh convergence analysis was performed based on the maximum displacements of intact humerus bone and von Mises stresses of wires. The refinement of mesh sizes was carried on until the changes in the results reached below 3%. In numerical simulations, each model included an average of 690,000 of 10-node quadratic tetrahedral (C3D10) elements with a maximum edge length of 1.4 mm (Fig. 3c).

### Biomechanical testing

**Fig. 4:**
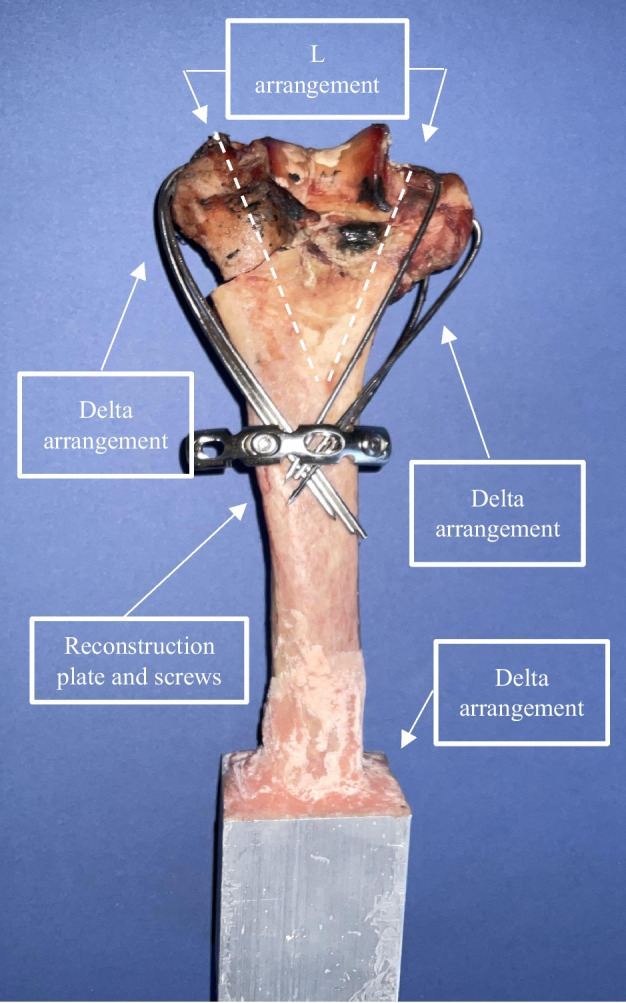
Configuration of implants on the cadaveric specimen.

A fresh frozen distal humerus of a human cadaver was harvested from a male donor at the age of 41, and mechanical properties of humerus bone were estimated from the age and sex of the donor [27, 28]. The specimen was stored in a freezer at − 20 °C. After osteotomy creation and simulating intra-articular Y-shape fracture at the distal end, as stated in the numerical section, a combination of L and Delta arrangements was applied in a specimen by a senior trauma surgeon. This procedure was performed with a set of 1.5 mm smooth K-wires that were bent after creating temporary fixation. The wires were then fixed under a four-hole reconstruction plate by cortical self-tapping screws that were screwed in pilot holes on the humerus shaft posteriorly. In the applied osteosynthesis, three wires had a Delta arrangement, and a couple of wires had an L arrangement with an angle of 60° with respect to the epicondylar axis. K-wires with the L method were cut just after egressed from distal fragments. Afterward, the specimen was proximally potted in an aluminum profile containing dental cement, as shown in Fig. 4. Different loading conditions were exerted on the distal fragment, using dental cement as a mold to apply compression and anterior bending.

**Fig. 5:**
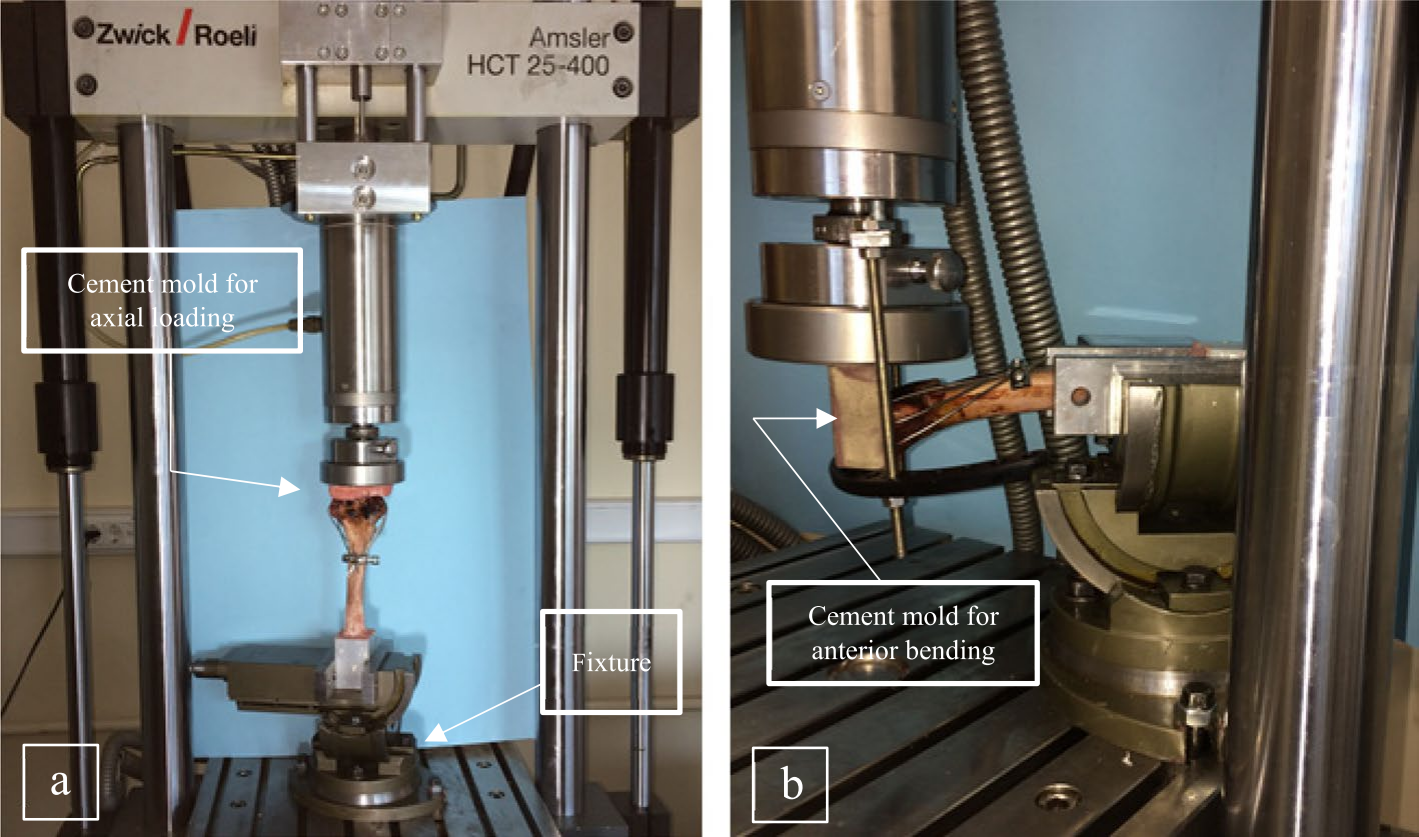
Biomechanical testing setup for (**a**) compression and (**b**) anterior bending.

To measure the stiffness, a cyclic loading in the range of 10 N to 100 N and 10 N to 30 N was applied in compression and bending, respectively [1, 4, 22, 24, 29]. At first, the specimen was subjected to axial loading, and finally, anterior bending was applied to cadaveric sample. A number of 20 cycles at 0.1 Hz was applied for both loading regimes consecutively [1, 4, 15, 24]. Biomechanical testing was performed by using a servo-hydraulic testing machine (Amsler HCT 25–400; Zwick/Roell AG, Germany). Testing setup for each loading condition is shown in Fig. 5.

### Research ethics committee approval

The use of cadaveric specimens in the biomechanical testing section was approved by Tehran University of Medical Sciences research ethics committee under the code IR.TUMS.MEDICINE.REC.1398.966.

## Results

### Finite element modeling

**Fig. 6:**
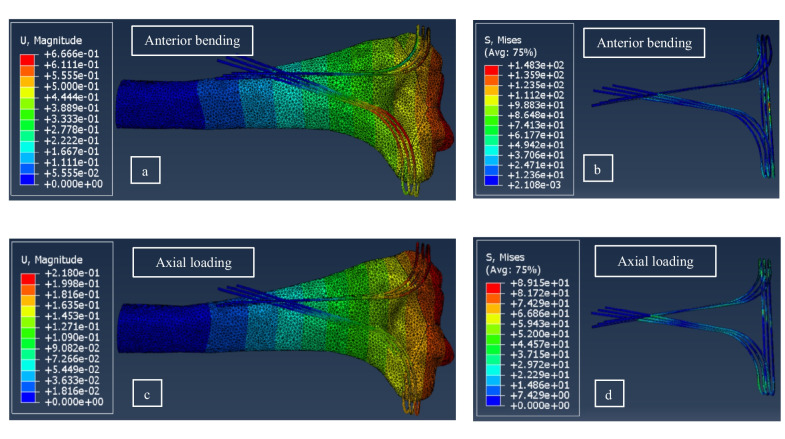
Displacement and von Mises contours for run number 7 of Table 3: (**a**) displacement under anterior bending, (**b**) von Mises stress under anterior bending, (**c**) displacement under axial loading, and (**d**) von Mises stress under axial loading. The displacement and stress values are in mm and MPa, respectively.

In the Delta configurations, wires in the anterior and distal areas of fragments experienced the maximum stresses. The maximum displacement values of fragments under different loading directions are listed in Table 3. In all models under different loading conditions, the maximum displacement appeared in the radial column or lateral fragments and the highest stresses in the bone occurred near the wire holes. Detailed results for Delta configuration are reported in Appendix A (Table 1A). Fig. 6a-d show displacement and von Mises stress contours for the run order number 7 under anterior bending and axial loading conditions.

**Table 3:** Maximum displacements of Delta models under anterior bending and axial loading

Run order	Numbers of wire	Diameter of wires (mm)	Plate height (mm)	Maximum displacement of fragments under a 100 N Axial load (mm)	Maximum displacement of fragments under a 30 N Bending load (mm)
1	1	1	55	**0.299**	**0.915**
2	1	1.5	65	**0.265**	**0.866**
3	1	2	75	**0.222**	**0.759**
4	2	1	65	**0.252**	**0.752**
5	2	1.5	75	**0.230**	**0.628**
6	2	2	55	**0.170**	**0.500**
7	3	1	75	**0.220**	**0.670**
8	3	1.5	55	**0.190**	**0.478**
9	3	2	65	**0.145**	**0.294**

**Fig. 7:**
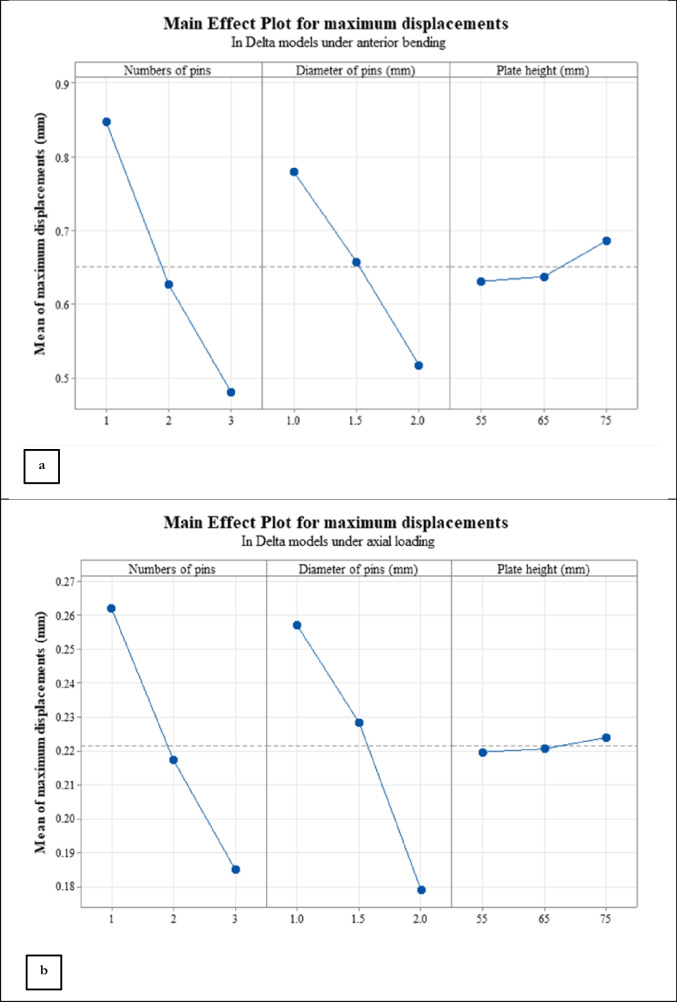
Main effect plots for the Delta models under (**a**) anterior bending and (**b**) axial loading.

Main effect plots for different influential parameters in Delta arrangement are illustrated in Fig. 7; under anterior bending loading, the number of wires had a more important influence on the stiffness of fixation with respect to the effect of diameter of wires as (see Fig. 7a). However, the diameter of wires under axial loading had a slightly larger effect on stability compared to the number of wires (Fig. 7b). The height of the reconstruction plate had a significantly less influence on the rigidity of construct with respect to other parameters under different loading conditions as seen in Fig. 7a and b.

**Fig. 8:**
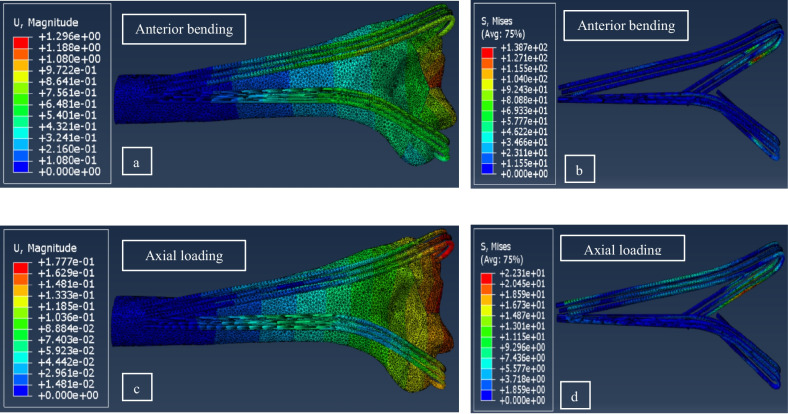
Displacement and von Mises contours for run number 15 of Table 4: (**a**) displacement under anterior bending, (**b**) von Mises stress under anterior bending, (**c**) displacement under axial loading, and (**d**) von Mises stress under axial loading. The displacement and stress values are in mm and MPa, respectively.

**Fig. 9:**
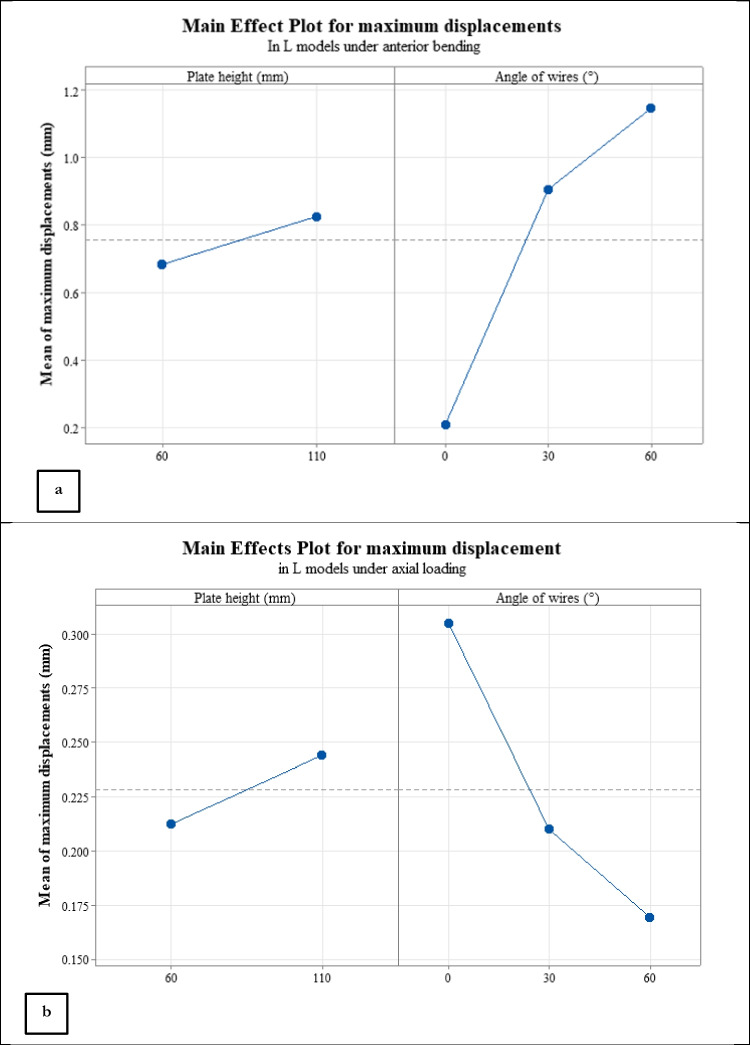
Main effect plots for the L models under (**a**) anterior bending and (**b**) axial loading.

Due to displacement magnitudes listed in Table 4, the L method yielded a lower stability under anterior bending in comparison to Delta simulations. Displacement and von Mises stress contours for model No. 15 under compression are shown in Fig. 8. Also, the Main effect plots for two influential parameters in the L method are shown in Fig. 9a and b; similar to the results of Delta configurations, plate height had only a small effect on stability, while the angle of pins in bending showed a significant impact on the fixation stiffness such that the stiffness in both loading conditions reduced with increasing this angle.

**Table 4:** Maximum displacements of the L models in bending and compressive force

Run Order	Angle of wires (°)	Plate height (mm)	Maximum displacement of fragments under a 100 N Axial load (mm)	Maximum displacement of fragments under a 30 N Bending load (mm)
10	0	60	**0.280**	**0.203**
11	0	110	**0.330**	**0.217**
12	30	60	**0.195**	**0.849**
13	30	110	**0.225**	**0.964**
14	60	60	**0.162**	**0.998**
15	60	110	**0.177**	**1.296**

### Biomechanical testing and validation of model

**Fig. 10:**
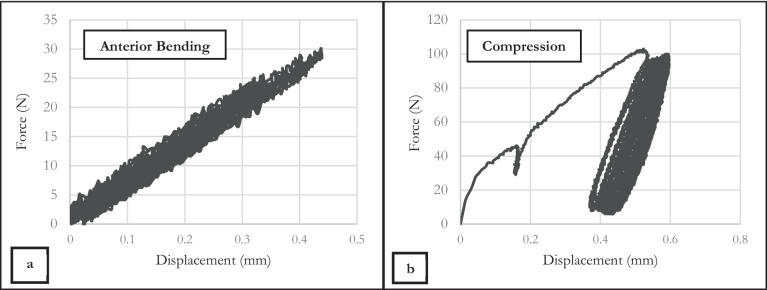
Load–displacement graphs of specimen under (**a**) anterior bending and (**b**) axial loading.

The cadaveric specimen (prepared as mentioned in Sect. 2.4) was subjected to anterior bending and axial loading with the maximum displacement of fragments during cyclic loading recorded as 0.46 ± 0.02 and 0.15 ± 0.03 mm, respectively. Load – displacement graphs of both loading conditions are provided in Fig.10a and b.

**Fig. 11:**
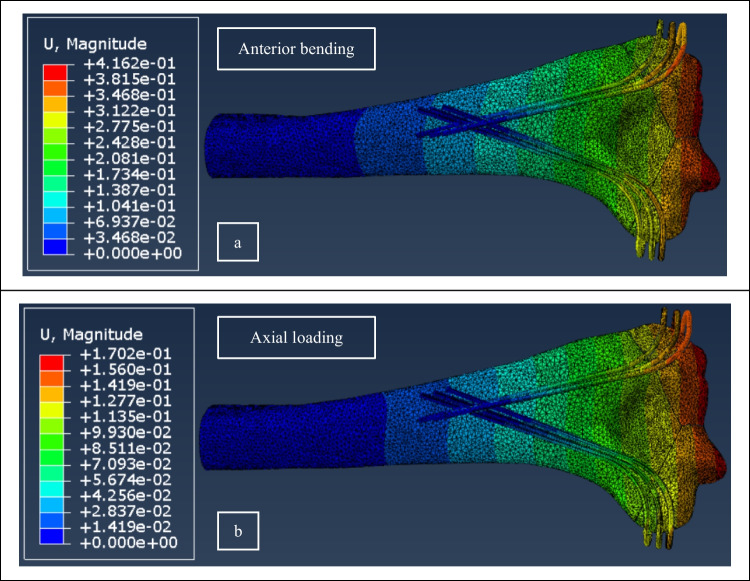
Displacement contours of the FE model built for validation with the cadaveric specimen under (**a**) anterior bending and (**b**) axial loading.

For validation purposes, an FE model was built in accordance with the fixation configuration of the cadaver specimen; i.e., with combination of a Delta configuration including three wires and an L arrangement with two 60-degree wires cut at the egression edge of fragments. The displacement contours of the validation model under anterior bending and axial loading are depicted in Fig. 11a and b, respectively. Comparing the results of FE simulation with those of the experimental testing, a difference of 13% in compression and 10% in bending was obtained. According to a small number of specimens and complexities in biomechanical testing, exceeding in limit errors are expected.

## Discussion

This study aimed to investigate biomechanical properties of different implant structures and configurations of the Persian fixation method under two different loading conditions. To date, a few studies [30] have been carried out to analyze biomechanical behavior of the pin and plate fixation method. This analysis inspects the biomechanical response of the Delta and L technique of this novel method in Y shape intra-articular fracture of distal humerus under pure compressive and anterior bending loads. In the present study, we attempted to use the acquired results to suggest recommendations that can be used in clinical practice. Loading conditions were designated according to the physiological environment and avoiding plasticity of a bone-implant construct.

We simulated fracture without placing a transverse gap at the fracture site which mimics real conditions for applying an osteosynthesis fixation. Most previous studies [1, 14, 16, 22–24], observed changes in the gap to acquire exact construct stiffness without interfering with bone surface contacts in load-bearing conditions. Therefore, it seems complicated to compare the result of this study with those which used the conventional plate and screw fixation method. However, it seems the pin and plate method presented a lower stability [14, 22, 23, 31] compared to the conventional method, but the stiffness of this novel method can become closer to that of the plate and screw configuration by choosing proper K-wire geometries and arrangement. Although, a quantitative comparison between obtained results in this study and previous studies is not feasible because many factors such as testing procedure, loading conditions, bone density of specimens, and type of sample should be considered. Moreover, since using bulky plates and screws is not efficient for stabilizing the highly osteoporotic bone and to stabilize small distal fragments, a Persian fixation can be an appropriate alternative for treatment in such cases because this method provides unique advantages that other methods would not present. Furthermore, this technique can be combined with standard methods to achieve a stable fixation in complex distal fractures. According to obtained results from two main different configurations of the Persian fixation method, the Delta arrangement provided more stiffness for the fixation construct compared to the L configuration and this difference was found to be more significant in bending load. The stiffness of L configuration can also be increased if insertion angle of the wire with respect to the epicondylar axis decreases. In both load cases analyzed, the height of the plate had a negligible effect on stability. In axial loading, the diameter of the pin had the most significant effect on stiffness, but in the anterior bending loading, the number of pins showed a larger impact for the Delta structure. It should be noted that raising the number of wires with small diameter has a greater risk of failure than using fewer wires with greater diameter. For example, in Table. 3, one K-wire with a dimeter of 2 mm produced almost the same stability as a two-pin construct with 1 mm diameter, while the maximum stress in the wires reduced by about 15% decreasing the risk of implant failure.

From a clinical point of view, most of the patients treated with the Persian fixation technique had a good ROM [19], and the fracture healing process was satisfactory in the subjects tracked [19]. One Distinctive feature of this technique is that it allows orthopedic surgeons to fix complex fractures and avoid using elbow prostheses. In addition, since the pin and plate method requires only K-wires and a small plate, treatment costs will be reduced compared to other fixation methods.

Similar to previous computational and biomechanical analyses dealing with the orientation and construction of osteosynthesis of distal humerus fractures, the present study includes some limitations. Replacing the constructional plate with completely constrained boundary conditions and assumption of an idealized interaction between K-wires and plate may not entirely represent real conditions beneath the plate. In addition, the plastic deformation of K-wires due to bending during surgery was not considered as a linear elastic behavior throughout the wire was assumed; i.e., similar to other studies that modeled K-wires. A more complex FE model would be then necessary for future research on this method. Applying other loading conditions, such as torsion and comparing with conventional methods in the numerical simulation should be verified by biomechanical testing, which was not feasible due to insufficient specimens.

## Conclusions

In this study, we attempted to examine the effects of surgical parameters on the stability of pin and plate fixation in distal humerus fractures using FEM and biomechanical testing. We took the first step to investigate the biomechanical properties of the Persian fixation method. We conclude that in the case of axial and anterior bending loading, the Delta method will be more stable and can yield a greater rigidity for osteosynthesis construct. Our finding shows that increasing the diameter of wires had superiority over the number of wires to achieve suitable rigidity since it significantly decreased the risk of failure. Also, we recommend in the case of L configuration, minimal insertion angle of wires with respect to epicondylar axis would offer a stable fixation in intra-articular fractures. It is important to note these deductions have been made for compression and anterior bending loading and other loading conditions have not been considered in this research.

## Supplementary information

**Additional file 1. **Detailed results of Delta and L models.

## Data Availability

All data generated or analyzed during this study are included in this published article and its supplementary information files.
