# Supplementary material for: Biomechanical analysis of different osteosynthesis configurations in the pin and plate fixation method for distal humerus fractures
Source: BMC Musculoskelet Disord. 2023 Jul 17;24:579. doi: 10.1186/s12891-023-06709-y (PMC10351186; doi:10.1186/s12891-023-06709-y)
Supplement: Supplementary file 1 — Additional file 1. Detailed results of Delta and L models. [file 12891_2023_6709_MOESM1_ESM.docx]

**Appendix A. Detailed results of Delta and L models**

Table 1A. Results of the Delta models under anterior bending and axial loading.

| Run order | Numbers of wire | Diameter of wires (mm) | Plate height (mm) | Maximum stress in the Bone under a 100 N Axial load (MPa) | Maximum stress in the Bone under a 30 N Bending load (MPa) | Maximum stress in the wires under a 100 N Axial load (MPa) | Maximum stress in the wires under a 30 N Bending load (MPa) | Maximum displacement of fragments under a 100 N Axial load (mm) | Maximum displacement of fragments under a 30 N Bending load (mm) |
| --- | --- | --- | --- | --- | --- | --- | --- | --- | --- |
| 1 | 1 | 1 | 55 | 85.1 | 256.1 | 125.3 | 293.1 | **0.299** | **0.915** |
| 2 | 1 | 1.5 | 65 | 47.8 | 123.2 | 103.1 | 239.4 | **0.265** | **0.866** |
| 3 | 1 | 2 | 75 | 36.7 | 86.4 | 69.3 | 136.7 | **0.222** | **0.759** |
| 4 | 2 | 1 | 65 | 65.5 | 157.2 | 133 | 222.2 | **0.252** | **0.752** |
| 5 | 2 | 1.5 | 75 | 50.8 | 73.2 | 93.9 | 101.5 | **0.230** | **0.628** |
| 6 | 2 | 2 | 55 | 29.1 | 49.3 | 52.7 | 70.75 | **0.170** | **0.500** |
| 7 | 3 | 1 | 75 | 34.4 | 119.8 | 89.2 | 148.3 | **0.220** | **0.670** |
| 8 | 3 | 1.5 | 55 | 24.2 | 66.4 | 78.2 | 85.18 | **0.190** | **0.478** |
| 9 | 3 | 2 | 65 | 17.7 | 31.8 | 39.1 | 59.13 | **0.145** | **0.294** |

| 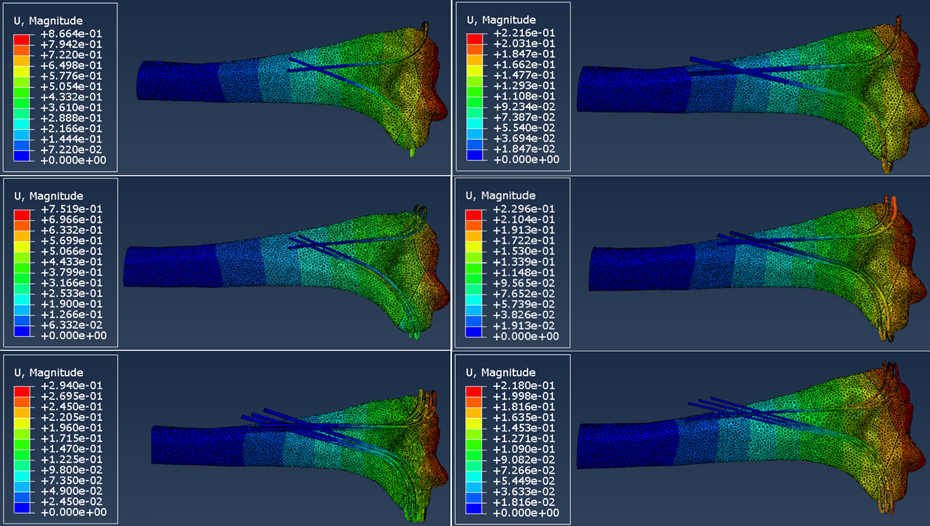 Anterior bending  a | 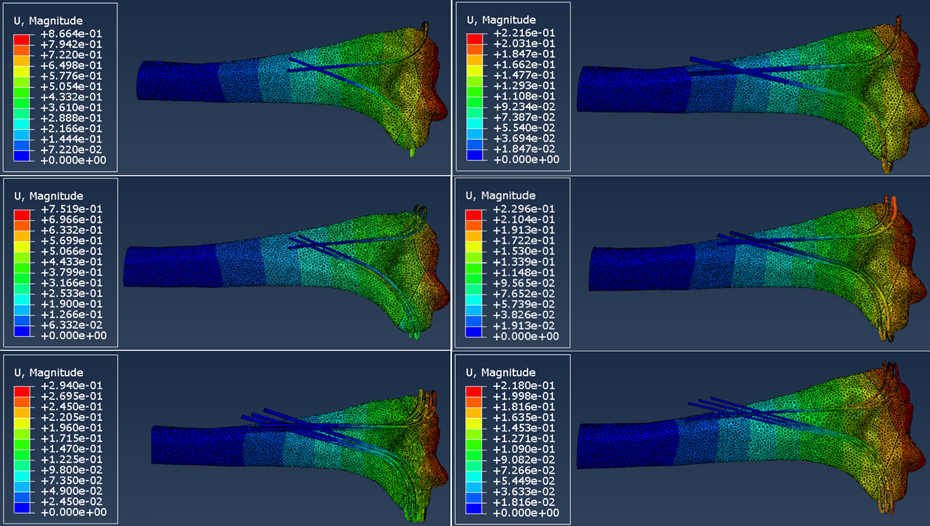 Axial loading  d |
| --- | --- |
| 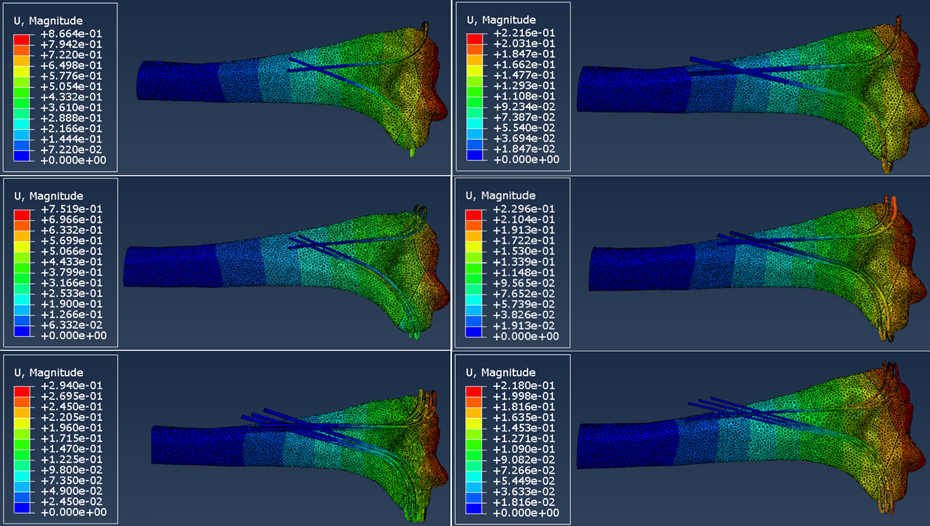 Anterior bending  b | 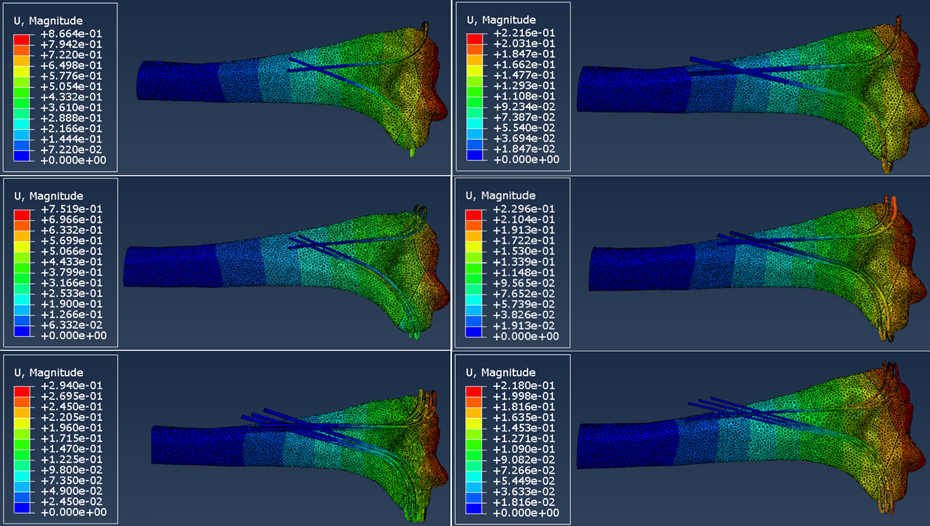 Axial loading  e |
| 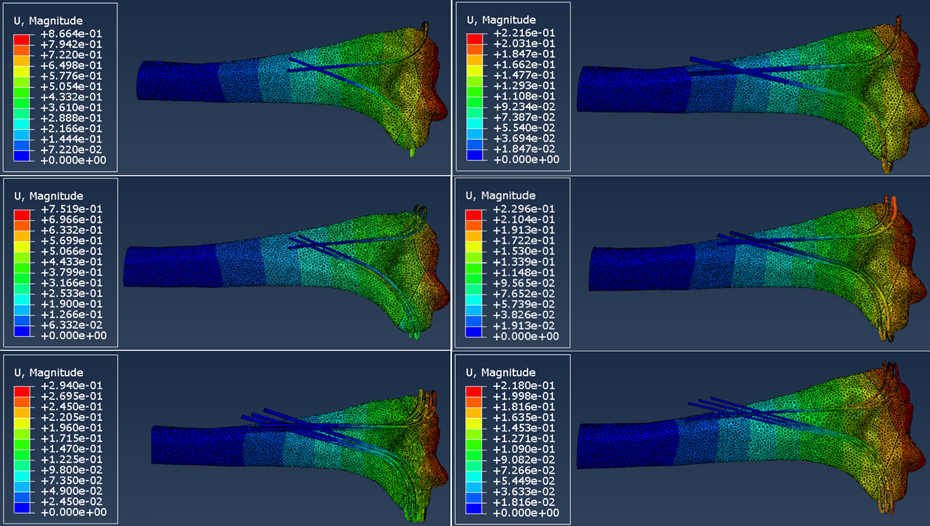 Anterior bending  c | 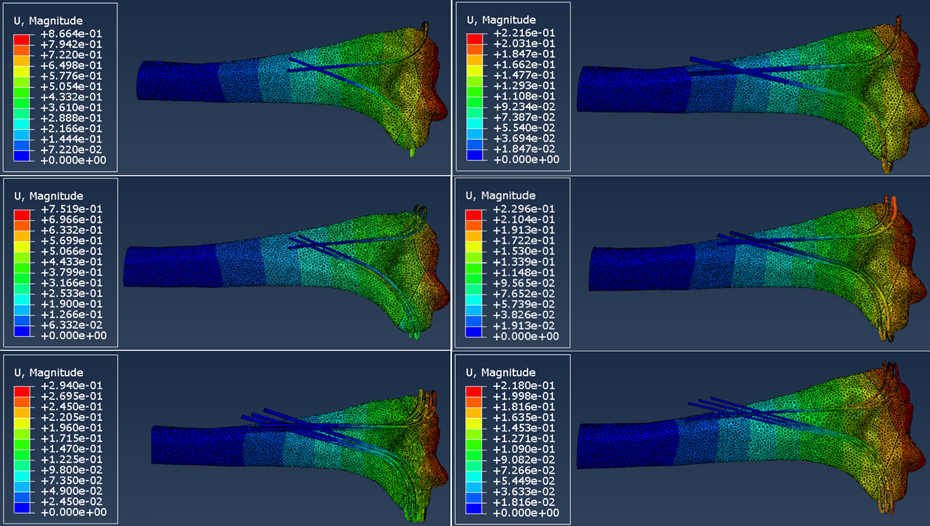 Axial loading  f |

**Fig. 1A.** Displacement contours of Delta models: (a-c) under anterior bending for run No. 2, 4 and 9, (d-f) under axial loading for run No. 3,5 and 7. The displacement values are in mm.

| 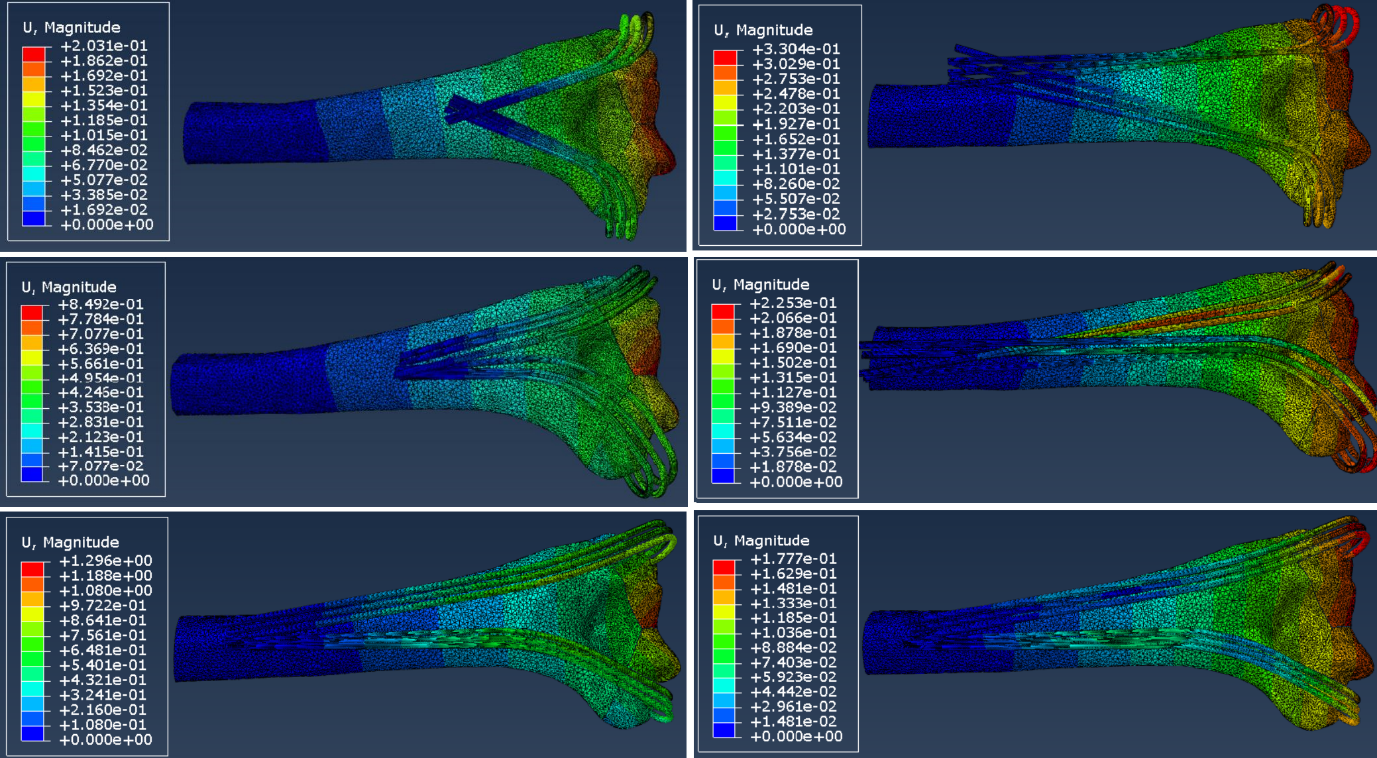 Anterior bending  a | 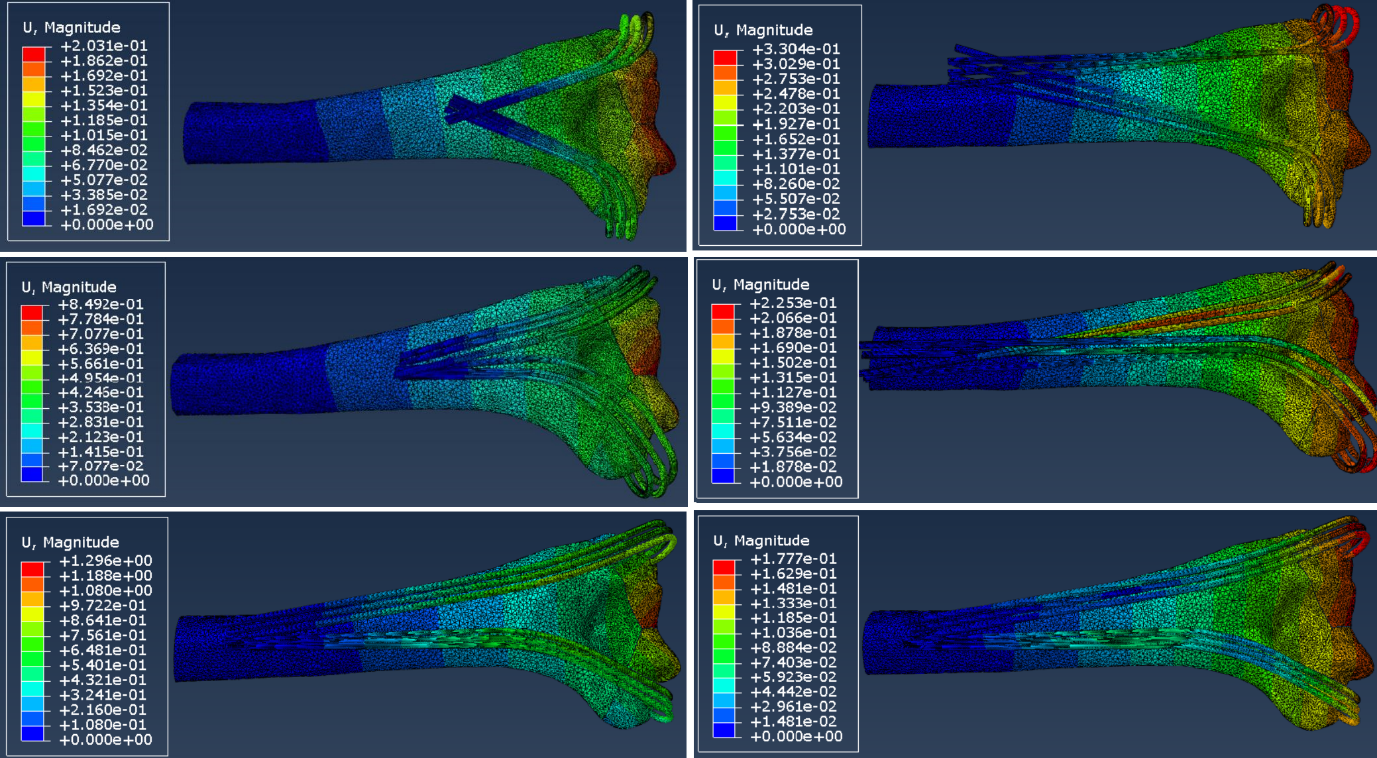 Axial loading  d loading |
| --- | --- |
| 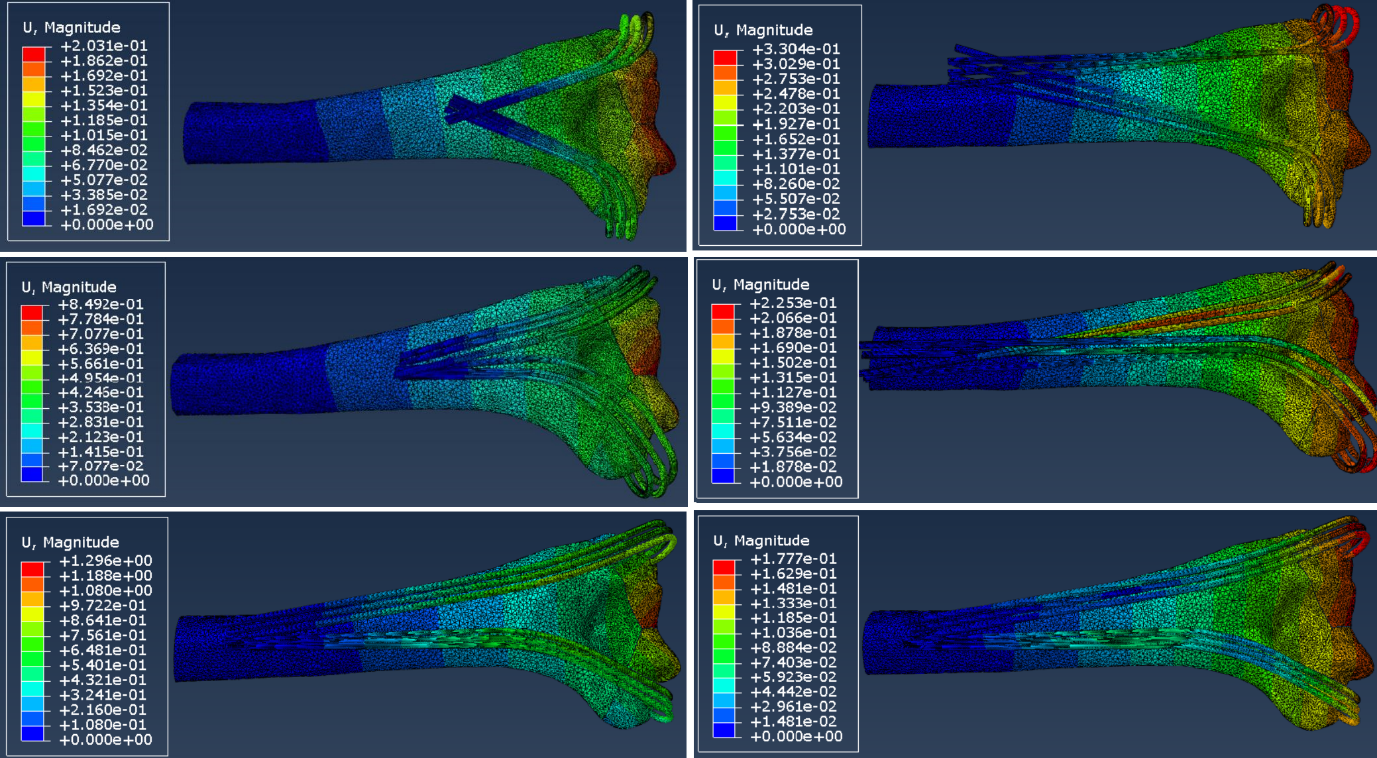 Anterior bending  b | 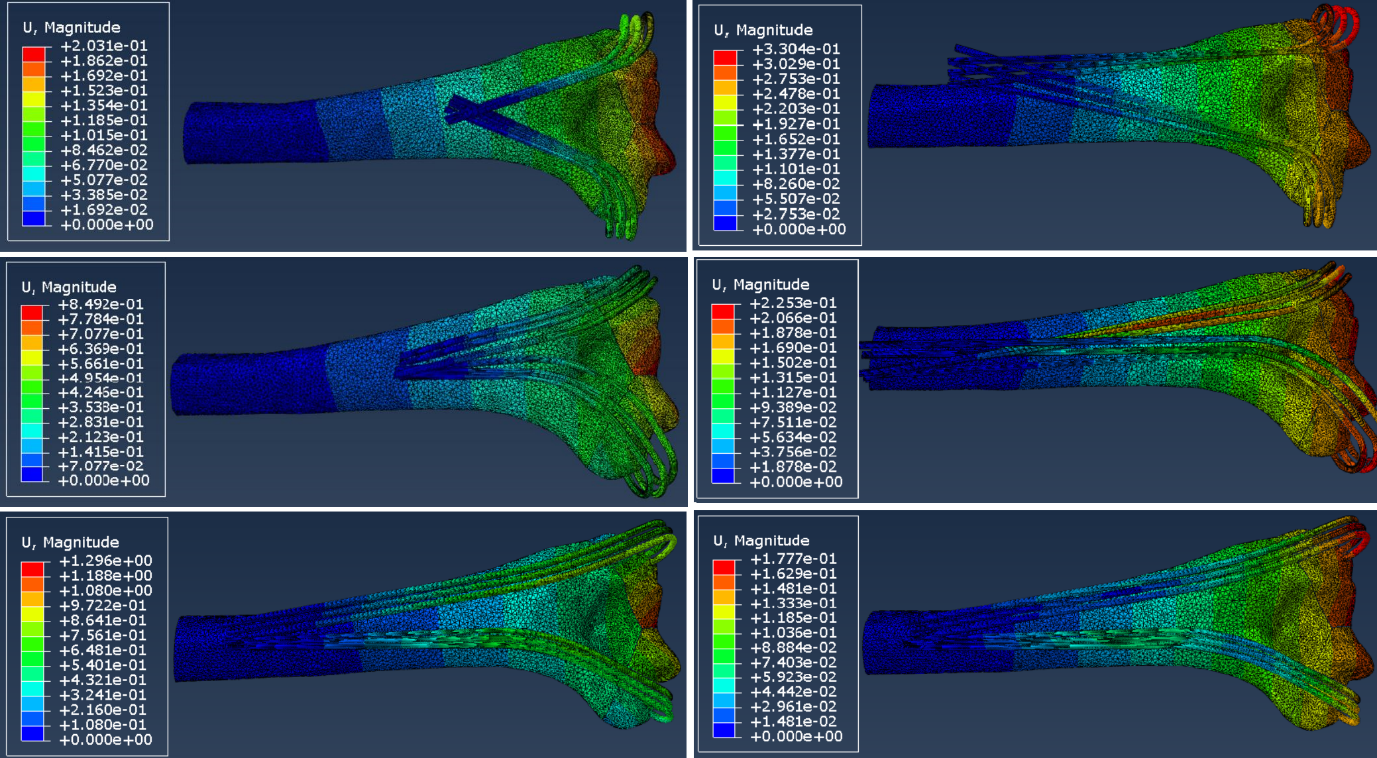 Axial loading  e |
| 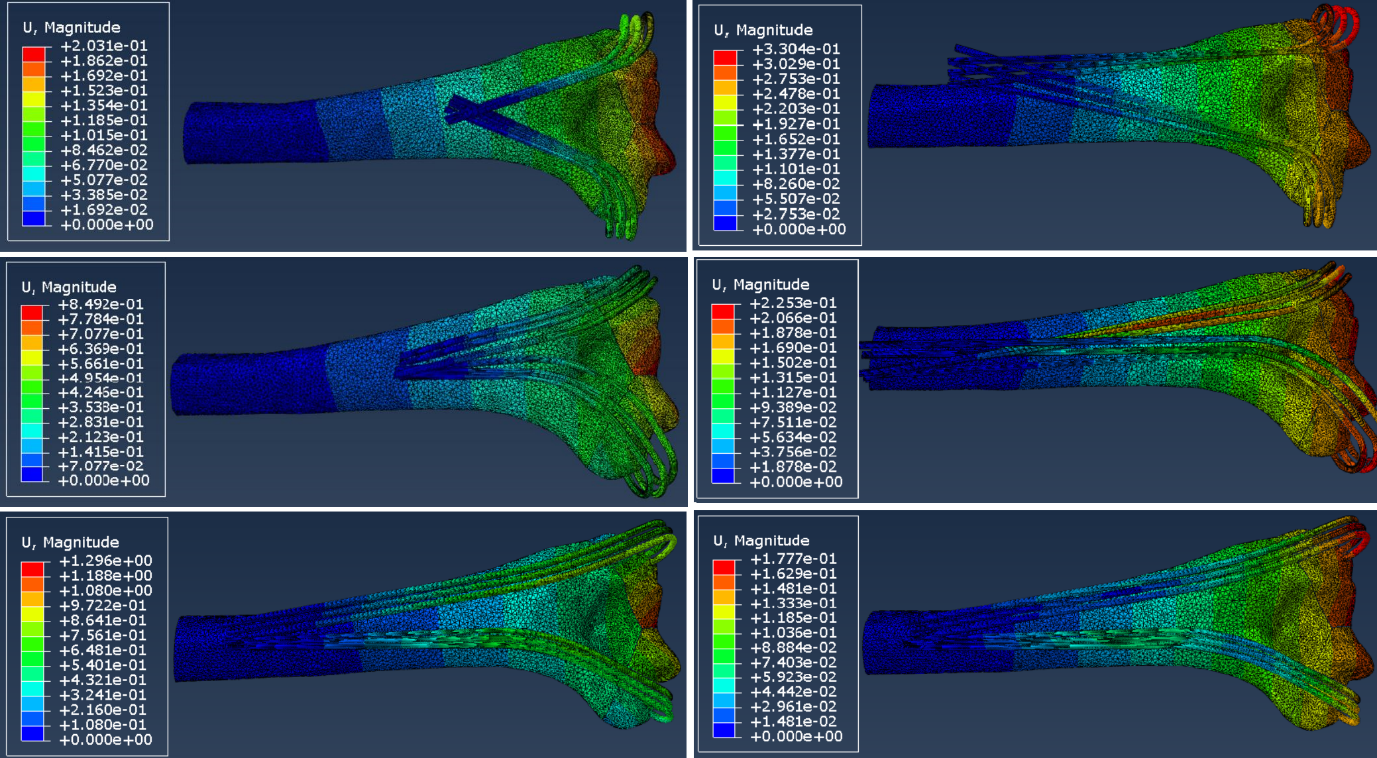 Anterior bending  c | 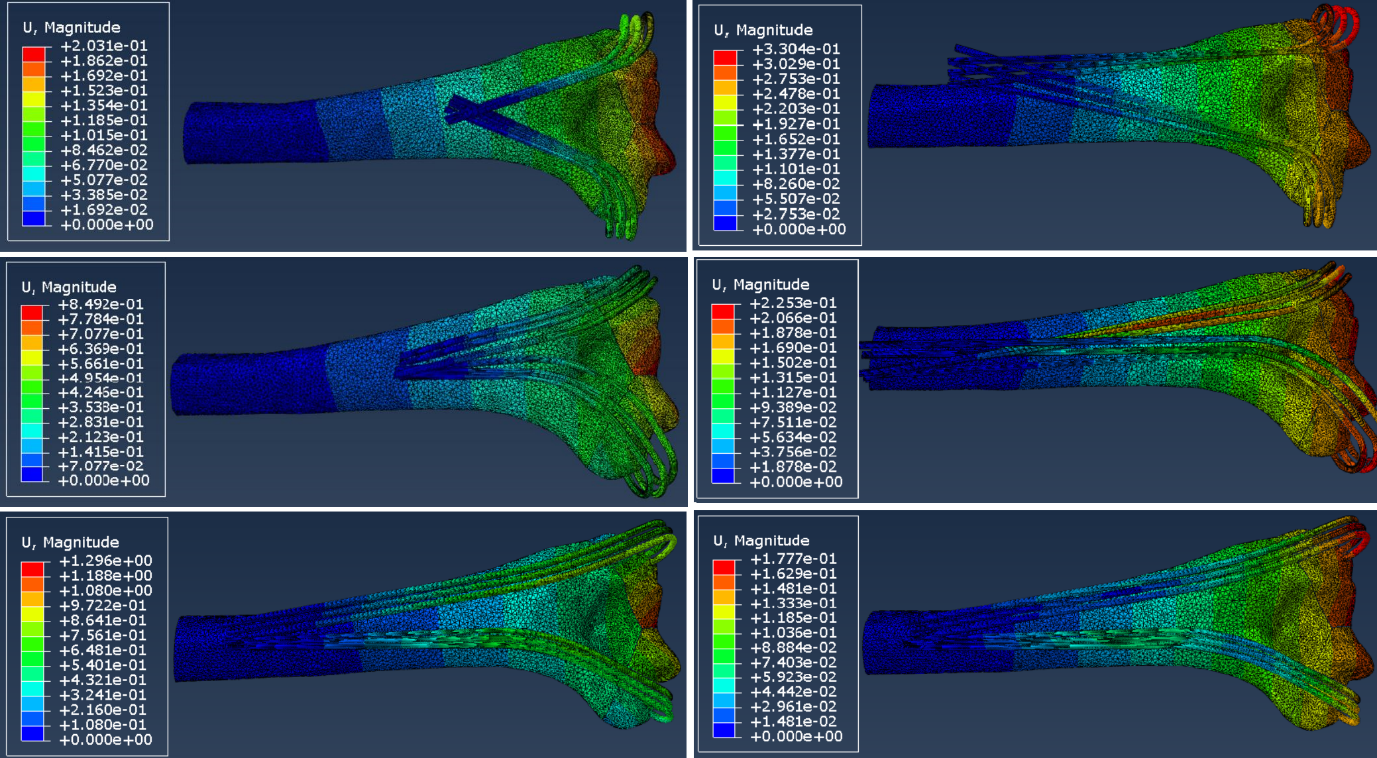 Axial loading  f |

**Fig. 2A.** Displacement contours of the L models: (a-c) under anterior bending for run No. 10, 12 and 15, (d-f) under axial loading for run No. 11,13 and 7. The displacement values are in mm.

Table 2A. Results of the L models in bending and compressive force.

| Run order | Angle of wires | Plate height (mm) | Maximum stress in the bone under a 100 N Axial load (MPa) | Maximum stress in the bone under a 30 N Bending Load (MPa) | Maximum stress in the wires under a 100 N Axial load (MPa) | Maximum stress in the wires under a 30 N Bending load (MPa) | Maximum displacement of fragments under a 100 N Axial load (mm) | Maximum displacement of fragments under a 30 N Bending load (mm) |
| --- | --- | --- | --- | --- | --- | --- | --- | --- |
| 10 | 0 | 60 | 82.5 | 37.7 | 26.4 | 141.6 | **0.280** | **0.203** |
| 11 | 0 | 110 | 47.2 | 44.9 | 23.2 | 62.6 | **0.330** | **0.217** |
| 12 | 30 | 60 | 57.2 | 71.4 | 35.4 | 127.5 | **0.195** | **0.849** |
| 13 | 30 | 110 | 43.3 | 79.7 | 26.3 | 58.6 | **0.225** | **0.964** |
| 14 | 60 | 60 | 41.8 | 104.6 | 29.9 | 114.5 | **0.162** | **0.998** |
| 15 | 60 | 110 | 25.6 | 117.8 | 22.4 | 138.7 | **0.177** | **1.296** |

| 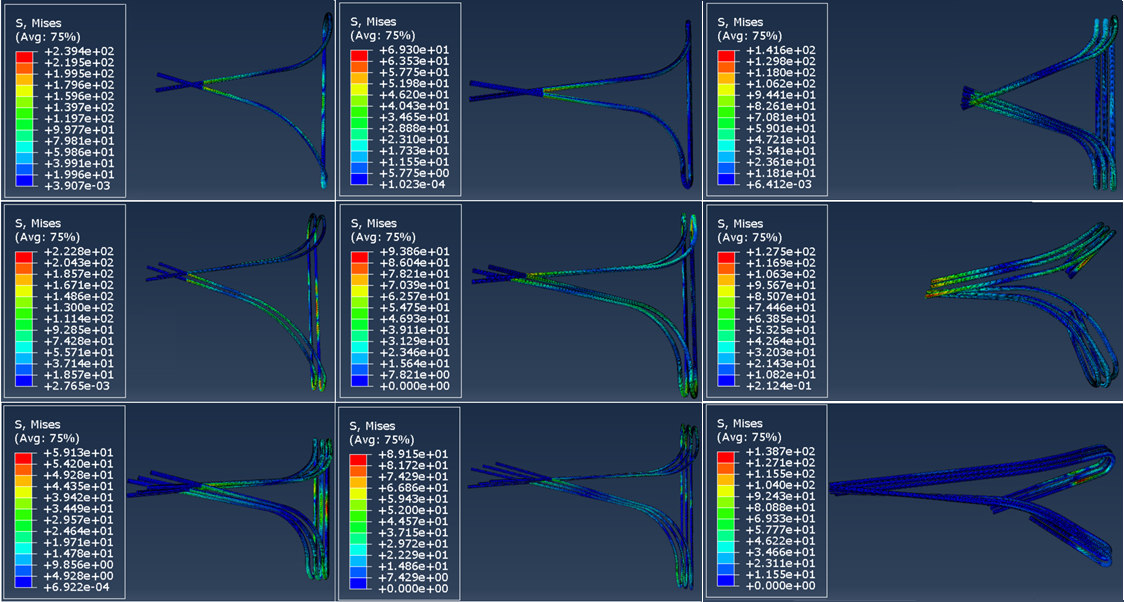 Anterior bending  a | 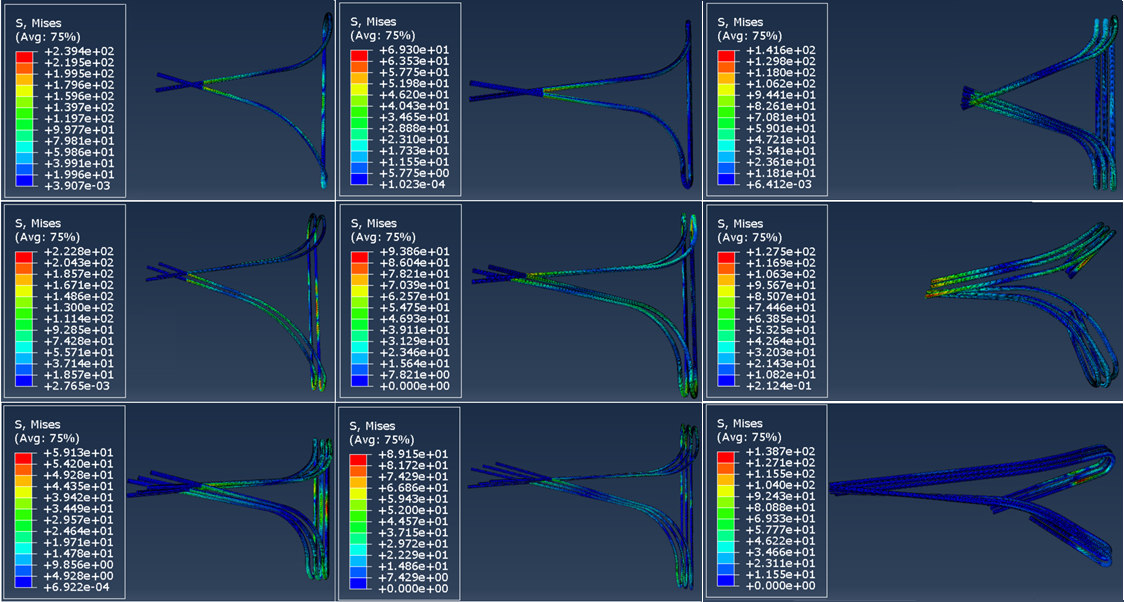 d  Axial loading | 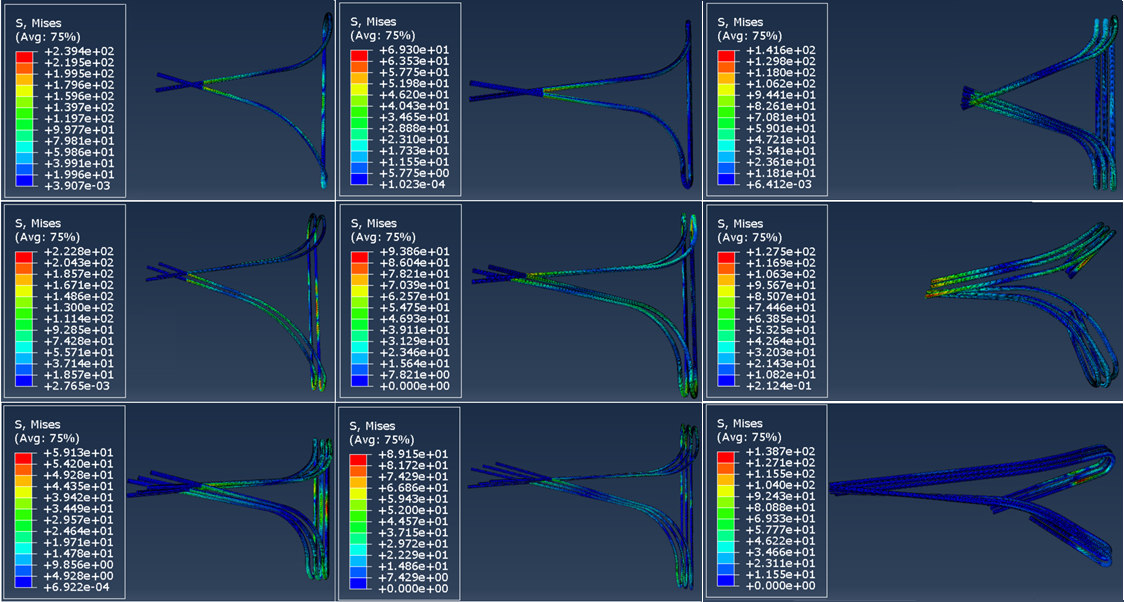 Axial loading  g |
| --- | --- | --- |
| 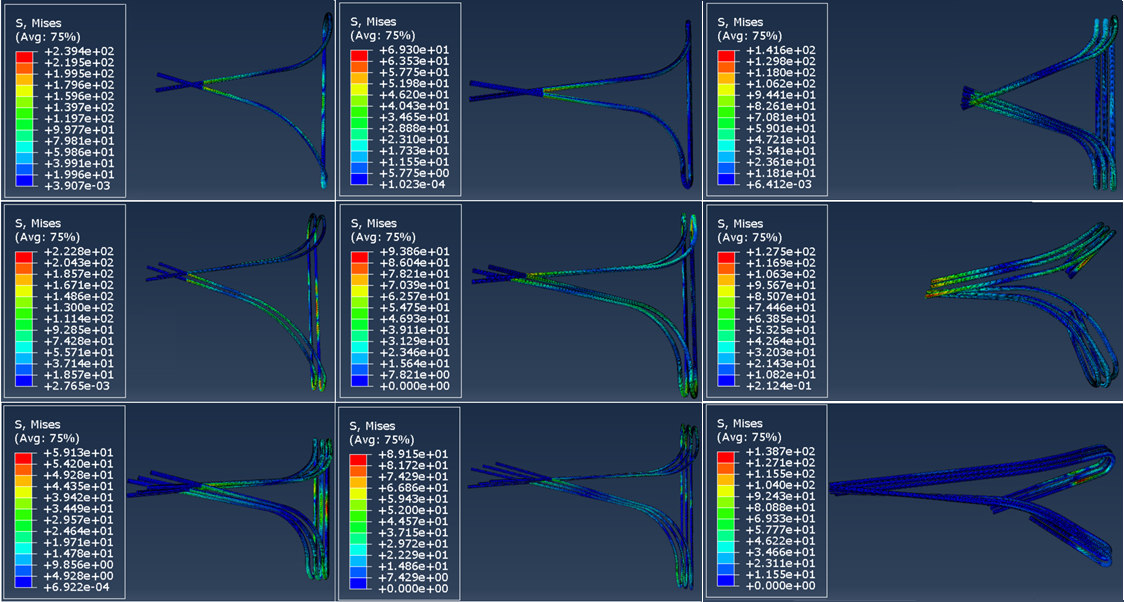 b  Anterior bending | 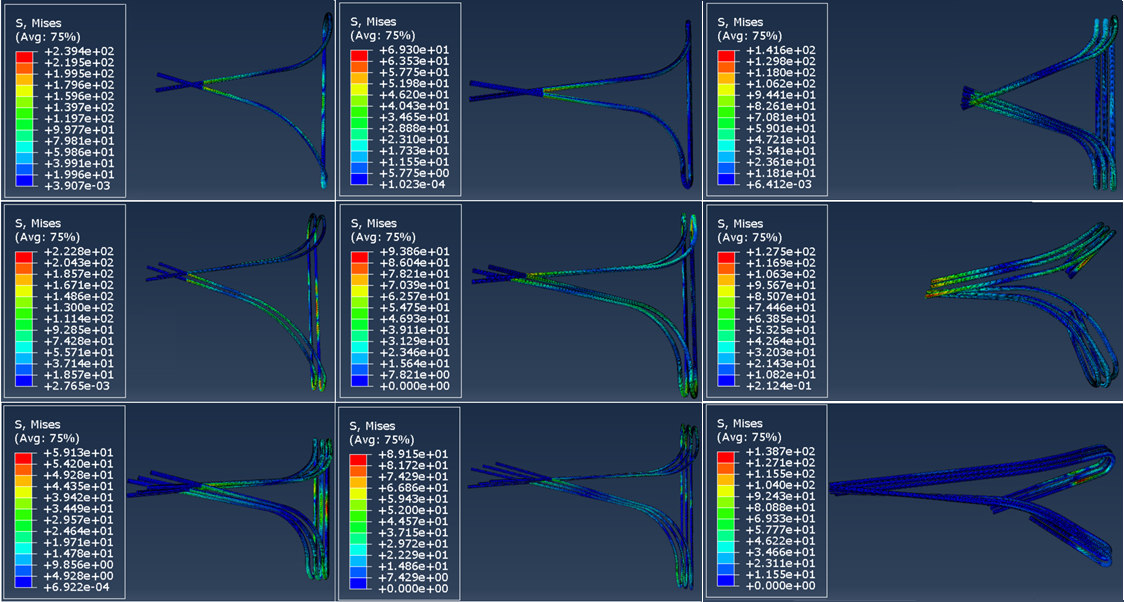 e  Axial loading | 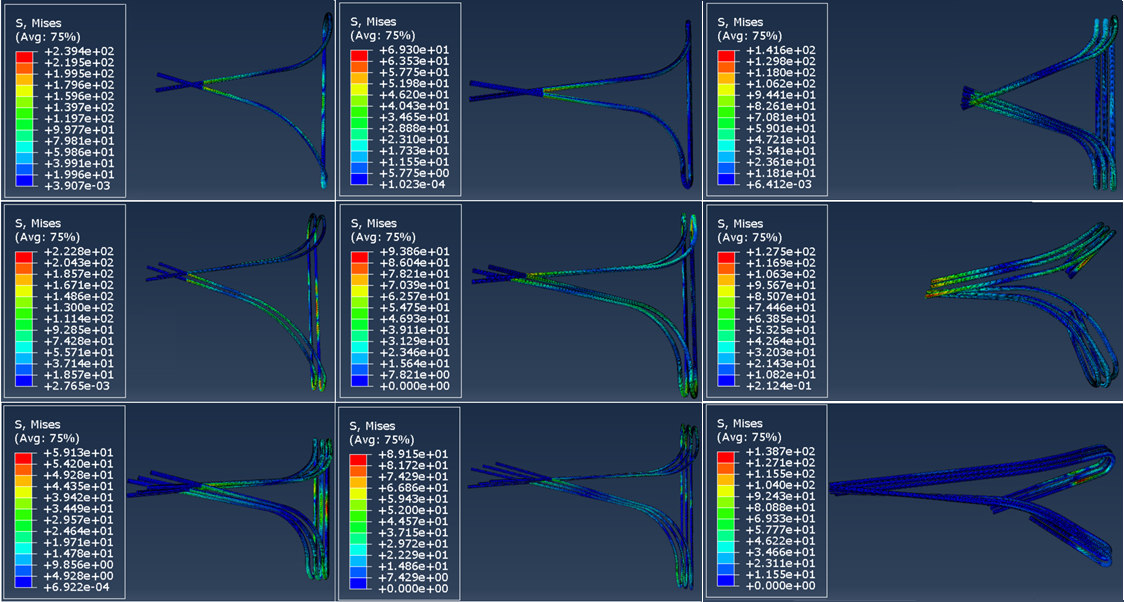 h  Anterior bending |
| 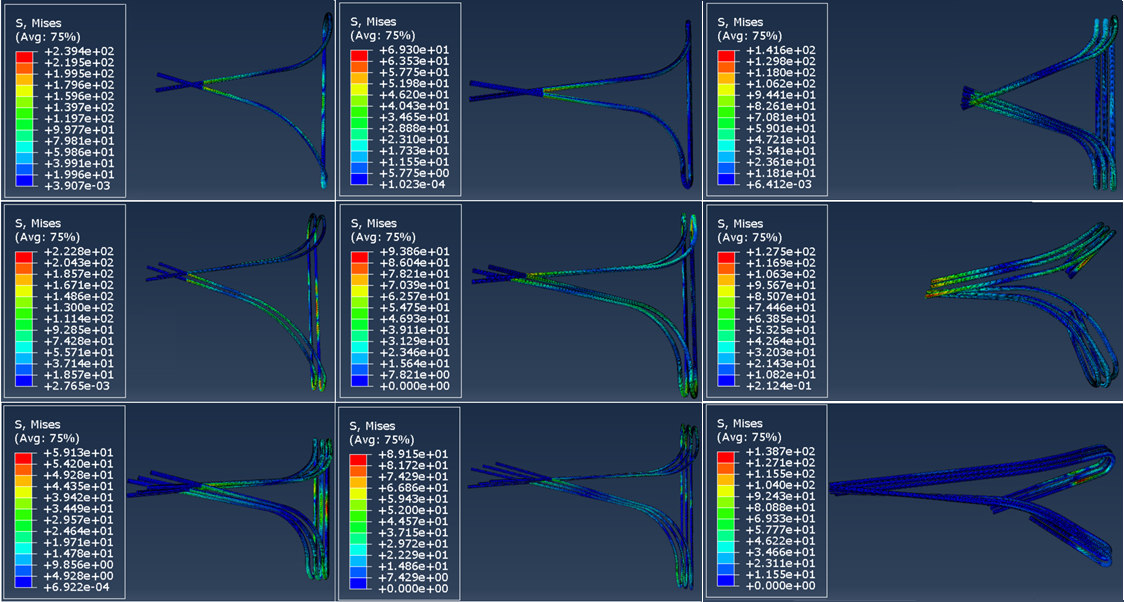 c  Anterior bending | 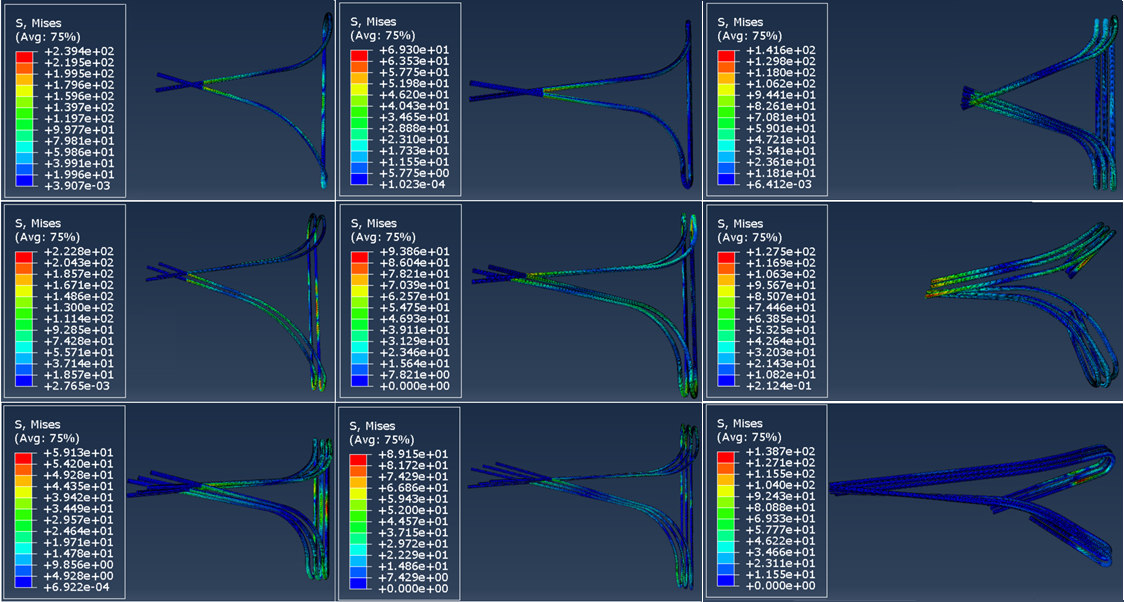 f  Axial loading | 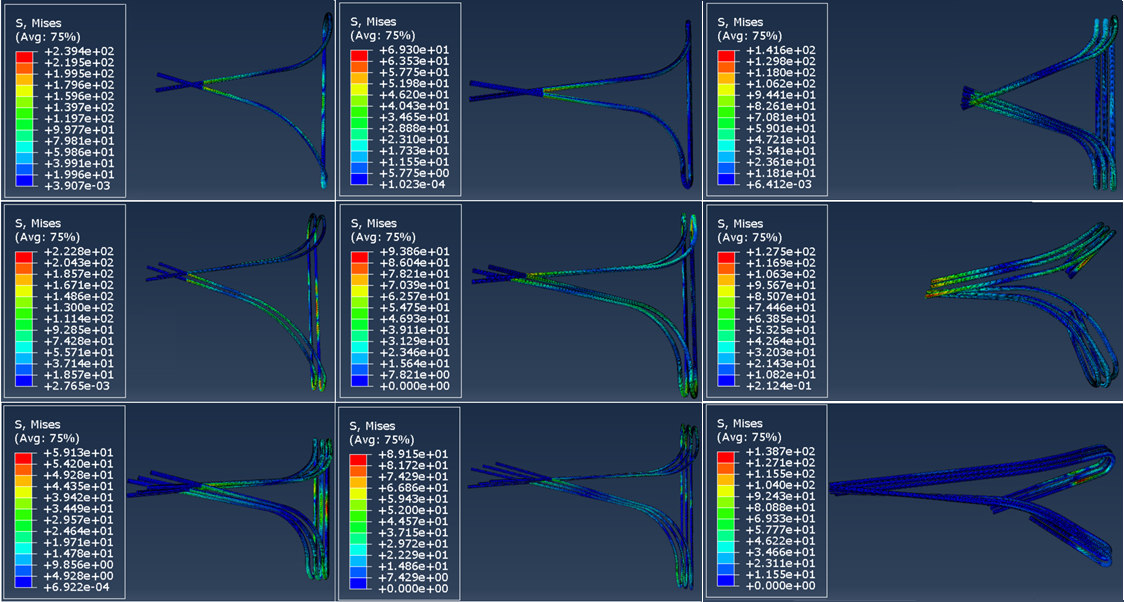 i  Anterior bending |

**Fig. 3A.** Von Mises stress contours of the L and Delta models: (a-c) under anterior bending for run No. 2,4 and 9, (d-f) under axial loading for run No. 3,5 and 7, (g-i) under anterior bending for run No. 10,12 and 15. The stress values are in MPa.
